# Geographic variations in end-of-life hospitalisations for patients with mental illness: a population-based observational study in England, UK

**DOI:** 10.1007/s44192-025-00252-z

**Published:** 2025-10-06

**Authors:** Emeka Chukwusa, Rebecca Wilson, Fiona Gaughran, Wei Gao

**Affiliations:** 1 Department of Palliative Care, Policy and Rehabilitation, King’s College London, Cicely Saunders Institute, Bessemer Road, Denmark Hill, London, SE5 9PJ UK; 2 Psychosis Studies, King’s College London, Institute of Psychiatry Psychology and Neuroscience, London, UK National Psychosis Service, South London and Maudsley NHS Foundation Trust, London, UK; 3 School of Public Health, Jiangxi Medical College, Nanchang University, Nanchang, China

**Keywords:** Mental illness, Geographic variations, Multiple end-of-life hospitalisations, End-of-life care

## Abstract

**Background:**

High rates of hospital admissions have been reported for patients with mental illness, but less is known about factors associated with multiple hospitalisations at the end-of-life in this group.

**Aim:**

To describe the geographical variations in end-of-life hospitalisations and examine factors associated with multiple hospitalisations (≥ 2) in the last 90 days of life for people with mental illness.

**Methods:**

A national population-based observational study in England UK using a linkage of Hospital Episode Statistics Admitted Patient Care (HES-APC) and the Office for National Statistics (ONS) death registry data. Our cohort comprised patients aged 18 and over, who died in England between 2018-04-01 and 2019-03-31 with HES-APC diagnoses of (1) Schizotypal, delusional disorders or schizophrenia, (2) schizoaffective or bipolar affective disorder, (3) substance use disorders; or (4) depressive episodes or recurrent depressive disorders. Geographic variations of end-of-life hospitalisations for each diagnostic group were described across National Health Services (NHS) regions. Modified Poisson regression models were used to estimate factors associated with multiple end-of-life hospitalisations in each diagnostic group.

**Results:**

A total of 49,775 patients with mental illness died in the year 2018–2019, of whom 50.2% (n = 25,004) had multiple end-of-life hospitalisation in the last 90 days of life. Factors positively associated with multiple end-of-life hospitalisations included older age, being resident in an urban area, cancer related deaths, and, for patients with depressive disorders, higher socioeconomic deprivation.

**Conclusion:**

Strengthening primary and community care services targeted at older adults with cancer could potentially reduce multiple end-of-life hospitalisations for patients with mental illness.

**Supplementary Information:**

The online version contains supplementary material available at 10.1007/s44192-025-00252-z.

## Introduction

Multiple hospitalisations at the end-of-life- also known as ‘burdensome or end-of-life transitions [1–3] - are associated with reduced quality of life for both patients and caregivers, increased healthcare costs, and a lower likelihood of dying in one’s preferred place of death [1]. Research has shown that people with mental illnesses are high impact users of health services [4] and they may have extended general hospital stays [5, 6], which contribute to rising healthcare costs [7]. Whilesome hospital admissions are necessary [8], others may be preventable or avoidable [9] potentially reflecting poor quality primary care and unmet healthcare needs [10, 11].

In the context of non-end of life admissions, the high rate of hospital admissions among patients with mental illness is linked with factors such as substance use disorders [12, 13], having two or more physical health conditions [14] or living alone or social isolation [15]. Variations in the rate of hospital admissions have been attributed to regional differences in the supply of health and social care [16] or a lack of quality community primary care provision [17, 18]. For example, in the US, Golberstein and colleagues, using Medicaid claims data, identified large regional variations in the use of mental health services among adult patients [19]. Factors associated with hospital admissions at the end-of-life are varied, including patient-related variables such as age, sex, ethnicity, and cause of death [20].

Multiple hospitalisations are common in end-of-life populations, yet few studies have examined factors associated with multiple end-of-life hospital admission for patients with mental illness. Some groups have looked at specific diagnostic groups at the end-of-life, such as dementia or patients with advanced cognitive and functional impairment [3], using data from a specific geographic area [14, 21]. For example, a recent study using routinely collected data comprising a cohort of patients with mental illness from South London found that hospitalisations in the last 90 days of life were common in patients with physical co-morbidities [14]. However, it is unclear whether this finding is accross other parts of England, UK.

 National level population-based studies exploring factors associated with multiple end-of-life hospitalisations in people with mental illness are limited. Knowledge of associated factors could provide health policy makers with valuable information on how to target unwarranted hospitalisations, thus improving care and reducing costs. We aimed to describe the geographical prevalence of multiple end-of-life hospitalisations and associated factors among a national cohort of patients with a mental illness in England, UK.

## Methods

### Study design settings and data source

We conducted a national population-based observational study involving data linkage of Hospital Episode Statistics (HES) Admitted Patient Care (APC) and the Office for National Statistics (ONS) death registry data. The HES-APC contains record of all admissions to NHS hospitals in England, including independent health providers paid for by the NHS [22]. Each row in the HES-APC database represents a single episode of care, which are combined into a spell—a spell begins when a patient is admitted and ends when a patient is discharged, transferred or dies [23]. The two datasets were deterministically linked through a unique patient encrypted identifier code (HESID), present in both datasets. The fully pseudonymised linked dataset was provided by NHS Digital.

The HES-APC and ONS linked dataset included demographic and clinical characteristics, hospitalisation details, and death records. Information on diagnosis on admission (primary and secondary diagnosis), ethnicity, year of birth, and Lower Super Out Area (LSOA) of residence were obtained from the HES APC database. LSOAs are geographic units with an average population of 1500 people. The data were further linked to the ONS postcode data file to derive patients’ clinical commissioning group (CCGs), region of residence, and Index of Multiple Deprivation (IMD) status. CCGs are administrative regions for planning and commissioning health and social care services in England [24]. The IMD is an area-based measure of socioeconomic status (SES) level across LSOAs in England. The IMD version 2011 was grouped into quintiles ranging from most (1) to least deprived (5). Regions were defined using the 2019, NHS England regional classification. There are seven NHS regions in England (East of England, London, Midlands, Northeast & Yorkshire, and Northwest, Southwest, and Southeast). Place of death, sex, cause of death, and number of contributory causes of death were derived from the ONS death records. Age was derived by calculating the difference in years between the year of birth and death.

### Study population

Patients aged 18 and over were included if their ONS registered date of death was between 2018-04-01 and 2019–03-31 and they had a diagnosis of mental illness recorded in the HES APC database between 2017-04-02 and 2019-03-31. The diagnoses were recorded according to the International Classification of Diseases 10th edition (ICD-10) codes. They comprised depressive episode (ICD-10: F32) or recurrent depressive disorder (ICD-10: F33), schizoaffective disorder or bipolar affective disorder (ICD-10), schizophrenia, schizotypal or delusional disorders (ICD-10: F20–F29) and substance use disorders (ICD-10: F10–19). Diagnoses of mental illness were derived through a hierarchical approach [25]. For example, if a patient has multiple diagnoses, any reference to a mental health diagnosis (in HES data) mentioned in Table 1 takes precedence and was assumed as their primary mental illness diagnosis.

**Table 1:** Characteristics of patients with mental illness who were admitted in the last 90 days of life

Variables	Level	Overall	Multiple admission in last 90 days of life	
			Yes	No
n		49,775	25,004 (50.2)^†^	24,771 (49.8)^†^
Gender (%)	Female	27,355 (55.0)	13,735 (54.9)	13,620 (55.0)
	Male	22,420 (45.0)	11,269 (45.1)	11,151 (45.0)
Age (mean (SD))		73.28 (15.42)	72.96 (14.8)	73.60 (16.02)
Age group (%)	18–54	6560 (13.2)	3158 (12.6)	3402 (13.7)
	55–64	6827 (13.7)	3657 (14.6)	3170 (12.8)
	65–74	10,092 (20.3)	5467 (21.9)	4625 (18.7)
	75–84	12,584 (25.3)	6397 (25.6)	6187 (25.0)
	85 +	13,712 (27.5)	6325 (25.3)	7387 (29.8)
Cause of death (%)	Cancers	12,363 (24.8)	6614 (26.5)	5749 (23.2)
	CBDs	2678 (5.4)	1253 (5.0)	1425 (5.8)
	COPDs	4312 (8.7)	2456 (9.8)	1856 (7.5)
	CVDs	6701 (13.5)	2889 (11.6)	3812 (15.4)
	Neurological conditions	948 (1.9)	469 (1.9)	479 (1.9)
	Other deaths	22,773 (45.8)	11,323 (45.3)	11,450 (46.2)
Year of death (%)	2018	35,863 (72.1)	17,858 (71.4)	18,005 (72.7)
	2019	13,912 (27.9)	7146 (28.6)	6766 (27.3)
Place of death (%)	Elsewhere/ Other places	6353 (12.8)	2498 (10.0)	3855 (15.6)
	Home	4504 (9.0)	1613 (6.5)	2891 (11.7)
	NHS establishments	26,407 (53.1)	15,666 (62.7)	10,741 (43.4)
	Non-NHS establishments	12,511 (25.1)	5227 (20.9)	7284 (29.4)
Ethnicity (%)	Asian	1013 (2.0)	531 (2.1)	482 (1.9)
	Black/mixed/others	1165 (2.3)	596 (2.4)	569 (2.3)
	British (white)	42,206 (84.8)	21,207 (84.8)	20,999 (84.8)
	Irish (white)	528 (1.1)	285 (1.1)	243 (1.0)
	Not stated/not known	3603 (7.2)	1716 (6.9)	1887 (7.6)
	Others (white)	1260 (2.5)	669 (2.7)	591 (2.4)
Diagnosis (%)	Depressive episode or recurrent depressive disorder	31,983 (64.3)	15,130 (60.5)	16,853 (68.0)
	Schizoaffective disorder or bipolar affective disorder	2137 (4.3)	1259 (5.0)	878 (3.5)
	Schizotypal/Delusional disorders/Schizophrenia	3971 (8.0)	2072 (8.3)	1899 (7.7)
	Substance use disorders	11,684 (23.5)	6543 (26.2)	5141 (20.8)
Number of contributory causes of death (%)	0	9520 (19.1)	4598 (18.4)	4922 (19.9)
	1	11,357 (22.8)	5557 (22.2)	5800 (23.4)
	2	10,338 (20.8)	5391 (21.6)	4947 (20.0)
	3	7432 (14.9)	3920 (15.7)	3512 (14.2)
	4 +	11,128 (22.4)	5538 (22.1)	5590 (22.6)
Settlement (%)	Rural	4050 (8.1)	1930 (7.7)	2120 (8.6)
	Urban	45,725 (91.9)	23,074 (92.3)	22,651 (91.4)
Socioeconomic status (%)	1 (Most deprived)	13,629 (27.4)	7033 (28.1)	6596 (26.6)
	2	10,953 (22.0)	5560 (22.2)	5393 (21.8)
	3	9454 (19.0)	4813 (19.2)	4641 (18.7)
	4	8436 (16.9)	4113 (16.4)	4323 (17.5)
	5	7303 (14.7)	3485 (13.9)	3818 (15.4)
Regions (%)	East-of-England	5668 (11.4)	2909 (11.6)	2759 (11.1)
	London	5667 (11.4)	3085 (12.3)	2582 (10.4)
	Midlands	9909 (19.9)	5168 (20.7)	4741 (19.1)
	North-East & Yorkshire	8819 (17.7)	4337 (17.3)	4482 (18.1)
	North-West	7830 (15.7)	3782 (15.1)	4048 (16.3)
	South-East	7368 (14.8)	3580 (14.3)	3788 (15.3)
	South-West	4514 (9.1)	2143 (8.6)	2371 (9.6)
Number of admissions in last 90 days of life (Median [IQR])		2.00 [0.00, 3.00]	3.00 [2.00, 4.00]	0.00 [0.00, 0.00]

*CBD—Cerebrovascular diseases; COPD—Chronic obstructive pulmonary diseases; CVD—cardiovascular disease. †Values are based on are row percentages

### Variable definition

The primary outcome was a binary measure of multiple hospitalisations, indicating the presence of two or more hospital admissions (1) or one or fewer hospitalisations in the last 90 days of life (0). In line with previous studies, multiple hospitalisations were defined as having two or more admissions for any reason in the final months of life [2, 3]. Covariates included patients’ sex (male and female), age at death (18–54, 55–64, 65–74, 75–84, 85+), ethnicity (Asian, Black/Mixed/Others, White British, White Irish, White other), place of death, SES, settlement (urban and rural), underlying cause of death, and number of contributory causes of death.

The place of death, derived from the ONS data, was classified into four categories: Home (patients’ own residence), NHS communal establishments (e.g., NHS care homes, hospices, and hospitals) non-NHS communal establishments (e.g., non-NHSmanaged hospices, care homes, private hospitals) and elsewhere and other places (e.g., hostels, prisons, detention centres, public spaces). Place of death was only included in the descriptive analysis. Patients’ cause of death and number of contributory causes of death were identified using ICD-10 codes. The cause of death was classified into six groups: Cancers (ICD-10: C00–C97), Neurological conditions (ICD-10: G35–G37, G20, F02.3, G12), Chronic Obstructive Pulmonary Diseases: COPD (ICD-10: J40–J44, J47), Cardiovascular Diseases (ICD-10: I00–I52, I70–I99), Cerebrovascular Diseases: CBDs (ICD: G45–G46, I60–I69).

### Statistical analysis

Patients’ sociodemographic characteristics were described using frequencies and percentages. The number of hospital admissions in the last 90 days of life was aggregated to CCGs and regional variations for each mental illness diagnosis were mapped using GIS choropleth maps.

A modified Poisson regression [26] was used to measure the association between patients’ demographics and hospitalisation for each diagnosis of mental illness (depressive episode (ICD-10: F32) or recurrent depressive disorder (ICD-10: F33), schizoaffective disorder or bipolar affective disorder (ICD-10), schizophrenia, schizotypal or delusional disorders (ICD-10. F20–F29) and substance use disorders (ICD-10)). We constructed four multivariable models; each modelled the associations between patients’ sociodemographic characteristics and the presence of two or more hospitalisations (1) versus 0 or 1 hospitalisation (0). All explanatory variables were included in the model. The strength of the association was described with Proportional Ratios (PRs) and 95% Confidence Intervals (95% CIs). Sensitivity analysis examined factors associated with multiple hospitalisations in the last 30 days of life. All statistical analyses, including GIS maps, were conducted in R software version 4.1.2 [27].

## Results

### Patients’ demographic characteristics and end-of-life hospitalisations by NHS regions

Our study cohort included 49,775 patients who died between April 1, 2018 and March 31, 2019 had a recorded diagnosis of mental illness (Table 1). About 50.2% of the cohort had multiple end-of-life hospitalisations (n = 25,004, 50.2%). The mean age at death was 72.96 years (SD: 14.8), and the majority of those with multiple end-of-life hospitalisations were white British (n = 21,207, 84.8% of 25,004). Depressive or recurrent depressive disorder was the most common mental illness (n = 31,983, 64.3% of 49,775), followed by substance use disorders (n = 11,684, 23.5% of 49,775), schizotypal & delusional disorders or schizophrenia (n = 3971, 8.0% of 49,775), and schizoaffective & bipolar affective disorder (n = 2137, 4.3% of 49,775). There was a higher proportion of depressive disorders in those with no end-of-life hospitalisations (n = 16,853, 68% of 24,771) compared to patients with multiple end-of-life hospitalisations (n = 15,130, 60.5% of 25,004). In terms of place of death, a significant proportion of those with multiple end-of-life hospital admissions died outside of home: NHS establishment: (n = 15,666, 62.7% of 25,004; non-NHS establishment: n = 5227, 20.9% of 25,004).

The median number of multiple end-of-life admissions across NHS regions (Table 2) was 3, with the highest in London (IQR:.00-5.00). Except for ethnicity and settlement (which were different in London from other regions), distributions of study population characteristic were generally similar across regions.

**Table 2:** Characteristics of study population with multiple end-of-life hospitalisation by NHS Regions

	level	Overall	East-of-England	London	Midlands	North-East Yorkshire	North-West	South-East	South-West
n		25,004	2909	3085	5168	4337	3782	3580	2143
Gender (%)	Female	13,735 (54.9)	1639 (56.3)	1619 (52.5)	2830 (54.8)	2406 (55.5)	2074 (54.8)	1987 (55.5)	1180 (55.1)
	Male	11,269 (45.1)	1270 (43.7)	1466 (47.5)	2338 (45.2)	1931 (44.5)	1708 (45.2)	1593 (44.5)	963 (44.9)
Age group (%)	18–54	3158 (12.6)	304 (10.5)	382 (12.4)	653 (12.6)	575 (13.3)	610 (16.1)	359 (10.0)	275 (12.8)
	55–64	3657 (14.6)	364 (12.5)	420 (13.6)	749 (14.5)	663 (15.3)	685 (18.1)	461 (12.9)	315 (14.7)
	65–74	5467 (21.9)	600 (20.6)	692 (22.4)	1136 (22.0)	983 (22.7)	856 (22.6)	756 (21.1)	444 (20.7)
	75–84	6397 (25.6)	771 (26.5)	809 (26.2)	1329 (25.7)	1112 (25.6)	883 (23.3)	919 (25.7)	574 (26.8)
	85 +	6325 (25.3)	870 (29.9)	782 (25.3)	1301 (25.2)	1004 (23.1)	748 (19.8)	1085 (30.3)	535 (25.0)
Underlying causes of death (%)	Cancers	6614 (26.5)	785 (27.0)	854 (27.7)	1320 (25.5)	1122 (25.9)	992 (26.2)	943 (26.3)	598 (27.9)
	CBDs	1253 (5.0)	157 (5.4)	154 (5.0)	267 (5.2)	217 (5.0)	171 (4.5)	184 (5.1)	103 (4.8)
	COPDs	2456 (9.8)	244 ((8.4)	292 (9.5)	481 (9.3)	494 (11.4)	399 (10.5)	352 (9.8)	194 (9.1)
	CVDs	2889 (11.6)	340 (11.7)	364 (11.8)	592 (11.5)	509 (11.7)	393 (10.4)	443 (12.4)	248 (11.6)
	Neurological conditions	469 (1.9)	63 (2.2)	58 (1.9)	104 (2.0)	78 (1.8)	53 (1.4)	82 (2.3)	31 (1.4)
	Other Deaths	11,323 (45.3)	1320 (45.4)	1363 (44.2)	2404 (46.5)	1917 (44.2)	1774 (46.9)	1576 (44.0)	969 (45.2)
Year of death (%)	2018	17,858 (71.4)	2071 (71.2)	2217 (71.9)	3679 (71.2)	3106 (71.6)	2737 (72.4)	2480 (69.3)	1568 (73.2)
	2019	7146 (28.6)	838 (28.8)	868 (28.1)	1489 (28.8)	1231 (28.4)	1045 (27.6)	1100 (30.7)	575 (26.8)
Place of death (%)	Elsewhere/Other Places	2498 (10.0)	322 (11.1)	313 (10.1)	495 (9.6)	429 (9.9)	391 (10.3)	307 (8.6)	241 (11.2)
	Home	1613 (6.5)	193 (6.6)	196 (6.4)	307 (5.9)	308 (7.1)	254 (6.7)	223 (6.2)	132 (6.2)
	NHS Establishments	15,666 (62.7)	1701 (58.5)	2008 (65.1)	3327 (64.4)	2688 (62.0)	2441 (64.5)	2206 (61.6)	1295 (60.4)
	Non-NHS Establishments	5227 (20.9)	693 (23.8)	568 (18.4)	1039 (20.1)	912 (21.0)	696 (18.4)	844 (23.6)	475 (22.2)
Ethnicity (%)	Asian	531 (2.1)	37 (1.3)	211 (6.8)	151 (2.9)	44 (1.0)	44 (1.2)	41 (1.1)	3 (0.1)
	Black/Mixed/Others	596 (2.4)	34 (1.2)	324 (10.5)	94 (1.8)	38 (0.9)	45 (1.2)	41 (1.1)	20 (0.9)
	British(White)	21,207 (84.8)	2498 (85.9)	1910 (61.9)	4455 (86.2)	3977 (91.7)	3449 (91.2)	3028 (84.6)	1890 (88.2)
	Irish(White)	285 (1.1)	29 (1.0)	109 (3.5)	57 (1.1)	12 (0.3)	35 (0.9)	26 (0.7)	17 (0.8)
	Not Stated/Not Known	1716 (6.9)	238 (8.2)	292 (9.5)	328 (6.3)	215 (5.0)	163 (4.3)	307 (8.6)	173 (8.1)
	Others(White)	669 (2.7)	73 (2.5)	239 (7.7)	83 (1.6)	51 (1.2)	46 (1.2)	137 (3.8)	40 (1.9)
Diagnosis (%)	Depressive episode or Recurrent depressive disorder	15,130 (60.5)	1902 (65.4)	1762 (57.1)	3244 (62.8)	2545 (58.7)	2012 (53.2)	2372 (66.3)	1293 (60.3)
	Schizoaffective disorder or Bipolar affective disorder	1259 (5.0)	185 (6.4)	168 (5.4)	227 (4.4)	206 (4.7)	168 (4.4)	172 (4.8)	133 (6.2)
	Schizotypal/Delusional disorders/Schizophrenia	2072 (8.3)	205 (7.0)	400 (13.0)	387 (7.5)	357 (8.2)	330 (8.7)	248 (6.9)	145 (6.8)
	Substance use disorders	6543 (26.2)	617 (21.2)	755 (24.5)	1310 (25.3)	1229 (28.3)	1272 (33.6)	788 (22.0)	572 (26.7)
Contributory causes of death (%)	0	4598 (18.4)	495 (17.0)	501 (16.2)	916 (17.7)	872 (20.1)	782 (20.7)	608 (17.0)	424 (19.8)
	1	5557 (22.2)	657 (22.6)	634 (20.6)	1095 (21.2)	918 (21.2)	959 (25.4)	739 (20.6)	555 (25.9)
	2	5391 (21.6)	631 (21.7)	663 (21.5)	1151 (22.3)	944 (21.8)	776 (20.5)	751 (21.0)	475 (22.2)
	3	3920 (15.7)	483 (16.6)	518 (16.8)	827 (16.0)	701 (16.2)	522 (13.8)	572 (16.0)	297 (13.9)
	4+	5538 (22.1)	643 (22.1)	769 (24.9)	1179 (22.8)	902 (20.8)	743 (19.6)	910 (25.4)	392 (18.3)
Settlement (%)	Rural	1930 (7.7)	371 (12.8)	4 (0.1)	477 (9.2)	259 (6.0)	108 (2.9)	407 (11.4)	304 (14.2)
	Urban	23,074 (92.3)	2538 (87.2)	3081 (99.9)	4691 (90.8)	4078 (94.0)	3674 (97.1)	3173 (88.6)	1839 (85.8)
Socioeconomic status (%)	1 (Most deprived)	7033 (28.1)	407 (14.0)	611 (19.8)	1543 (29.9)	1825 (42.1)	1858 (49.1)	436 (12.2)	353 (16.5)
	2	5560 (22.2)	628 (21.6)	1016 (32.9)	1099 (21.3)	887 (20.5)	718 (19.0)	706 (19.7)	506 (23.6)
	3	4813 (19.2)	756 (26.0)	639 (20.7)	1004 (19.4)	696 (16.0)	486 (12.9)	720 (20.1)	512 (23.9)
	4	4113 (16.4)	623 (21.4)	505 (16.4)	818 (15.8)	539 (12.4)	413 (10.9)	785 (21.9)	430 (20.1)
	5	3485 (13.9)	495 (17.0)	314 (10.2)	704 (13.6)	390 (9.0)	307 (8.1)	933 (26.1)	342 (16.0)
Number of admissions last 90 days (median [IQR])		3.00 [2.00, 4.00]	3.00 [2.00, 4.00]	3.00 [2.00, 5.00]	3.00 [2.00, 4.00]	3.00 [2.00, 4.00]	3.00 [2.00, 4.00]	3.00 [2.00, 4.00]	3.00 [2.00, 4.50]

*CBD, cerebrovascular diseases; COPD, chronic obstructive pulmonary diseases; CVD, cardiovascular disease

†Values are based on are row percentages

### Variations in end-of-life hospitalisations by diagnosis of mental illness

**Fig. 1:**
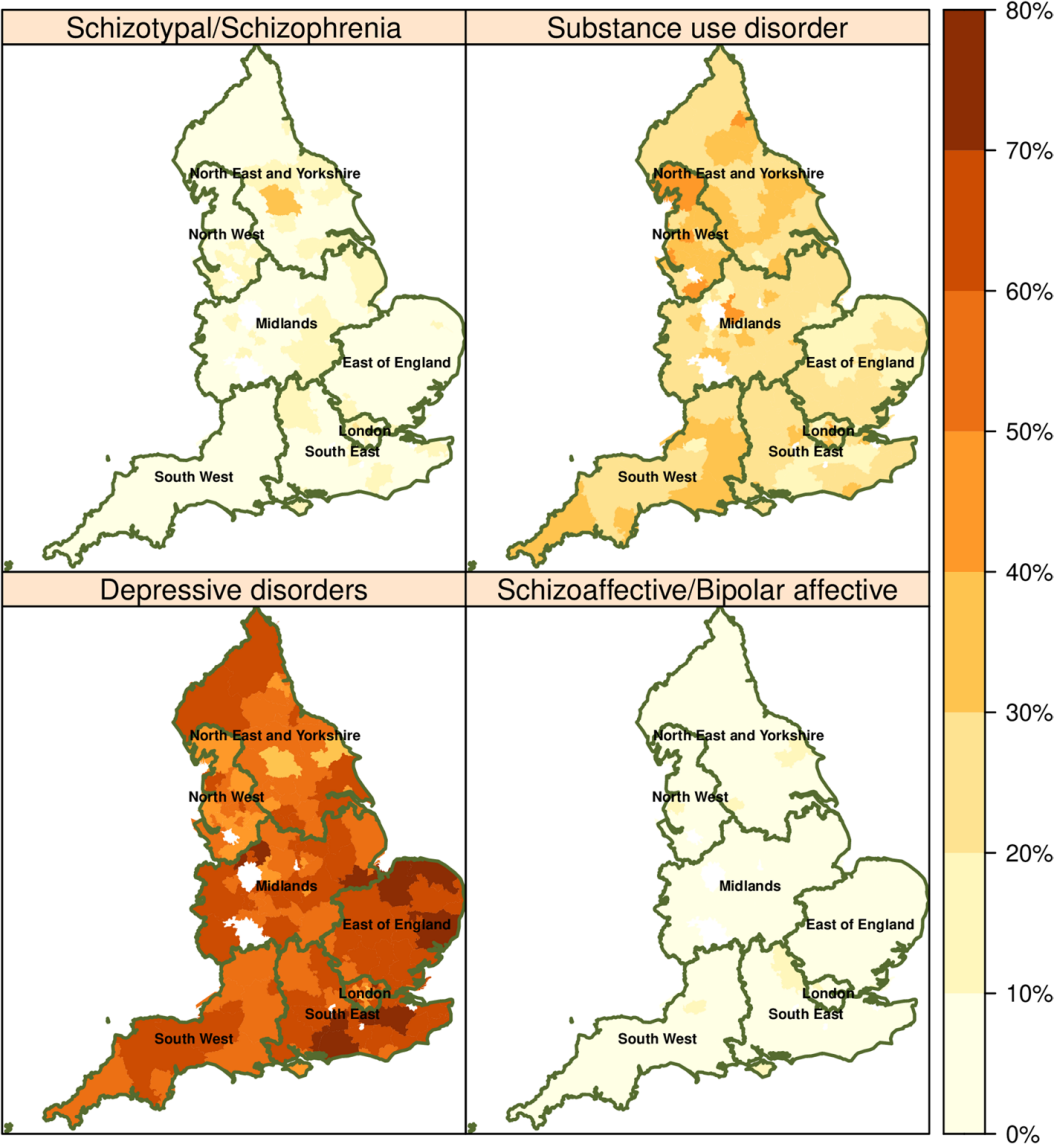
Geographic variations in end-of-life hospitalisations by diagnosis group. The digital boundary file contains OS data © Crown copyright and database right 2019

There were considerable regional variations in the percentage of patients who had multiple end-of-life hospitalisations across NHS regions (Fig. 1). This variation was more pronounced among patients diagnosed with substance use disorders or depressive disorders, whereas rates among those with a diagnosis of schizotypal & schizoaffective or bipolar disorder were evenly spread across NHS regions. Overall, multiple end-of-life hospitalisations for patients with a diagnosis of substance abuse disorders, schizotypal & schizoaffective disorder or bipolar were relatively low (less than 50%).

### Factors associated with multiple end-of-life hospitalisations in the last 90 days of life

Table 3 presents fully adjusted associations between covariates and end-of-life hospitalisations for each diagnosis. Older age (55+ compared to 18–54) was associated with an increased risk of multiple end-of-life hospitalisations, except for patients with substance use disorders aged 85 years and over. Residing in an urban area, increased the risk of multiple end-of-life hospitalisations across all diagnostic group. Patients with depressive or recurrent depressive disorder in socioeconomic quintiles 2 to 5, had a lower risk of multiple end-of-life hospitalisations compared to those in the most deprived quintile (quintile 1). In addition, patients with depressive or recurrent depressive disorder whose underlying cause of death was COPD had a higher risk of multiple end-of-life hospitalisations. In terms of contributory causes of death, having four or more contributory causes of death increased the risk of multiple end-of-life hospitalisations, except among those with substance use disorders.

**Table 3:** Factors associated with multiple end-of-life hospitalisations in the last 90 days of life by diagnosis of mental illness

Variables	Levels	Depressive episode or Recurrent depressive disorder	Schizoaffective disorder or Bipolar affective disorder	Schizotypal/Delusional disorders/Schizophrenia	Substance use disorders
		PR (95% CI)	PR (95% CI)	PR (95% CI)	PR (95% CI)
Gender	Female	Ref	Ref	Ref	Ref
	Male	0.98(0.96–1.00)	0.96(0.90–1.04)	0.95(0.89–1.01)	1.00(0.97–1.03)
Age group	18–54	Ref	Ref	Ref	Ref
	55–64	1.12(1.06–1.19)	1.39(1.17–1.65)	1.2(1.06–1.36)	1.08(1.03–1.13)
	65–74	1.16(1.1–1.22)	1.52(1.3–1.78)	1.26(1.13–1.42)	1.08(1.03–1.13)
	75–84	1.11(1.06–1.17)	1.51(1.29–1.76)	1.24(1.11–1.38)	1.05(0.99–1.1)
	85+	1.06(1.01–1.12)	1.39(1.18–1.63)	1.03(0.91–1.16)	0.96(0.88–1.04)
Ethnicity		Ref	Ref	Ref	Ref
	Black/mixed/others	0.95(0.84–1.07)	0.88(0.67–1.17)	0.92(0.78–1.09)	1.02(0.86–1.22)
	British (white)	1.01(0.93–1.1)	0.91(0.74–1.12)	0.92(0.8–1.05)	1.01(0.89–1.16)
	Irish (white)	1.04(0.91–1.19)	0.95(0.68–1.35)	0.92(0.69–1.21)	1.00(0.83–1.21)
	Not stated/not known	0.95(0.86–1.04)	0.85(0.67–1.09)	0.83(0.7–0.98)	0.99(0.86–1.14)
	Ethnicity others (white)	1.06(0.95–1.18)	0.86(0.64–1.16)	0.92(0.74–1.13)	1.04(0.89–1.22)
Number of Contributory causes of death	0	Ref	Ref	Ref	Ref
	1	1.05(1.01–1.09)	1.06(0.94–1.19)	1.02(0.92–1.13)	1.04(0.99–1.09)
	2	1.12(1.08–1.17)	1.19(1.06–1.35)	1.11(1.00–1.22)	1.13(1.07–1.19)
	3	1.15(1.1–1.19)	1.23(1.08–1.4)	1.15(1.03–1.28)	1.13(1.07–1.2)
	4+	1.12(1.07–1.16)	1.15(1.02–1.3)	1.13(1.02–1.25)	0.99(0.94–1.05)
Socioeconomic status	1 (Most deprived)	Ref	Ref	Ref	Ref
	2	0.96(0.93–1)	1.04(0.94–1.15)	0.98(0.91–1.06)	1.01(0.97–1.05)
	3	0.99(0.95–1.02)	1.05(0.95–1.17)	0.99(0.91–1.08)	1.01(0.96–1.06)
	4	0.95(0.92–0.99)	0.97(0.87–1.09)	0.93(0.84–1.03)	1.02(0.96–1.07)
	5	0.94(0.9–0.97)	0.95(0.83–1.07)	0.97(0.87–1.09)	1.00(0.94–1.06)
Settlement	Rural	Ref	Ref	Ref	Ref
	Urban	1.02(0.98–1.07)	1.12(0.97–1.29)	1.02(0.89–1.17)	1.04(0.96–1.12)
Underlying causes of death	Cancer	Ref	Ref	Ref	Ref
	*CBDs	0.89(0.84–0.94)	0.81(0.68–0.97)	0.89(0.77–1.01)	0.76(0.68–0.85)
	COPDs	1.03(0.98–1.07)	0.91(0.79–1.05)	0.97(0.86–1.08)	0.99(0.94–1.04)
	CVDs	0.79(0.76–0.83)	0.74(0.64–0.84)	0.69(0.62–0.78)	0.77(0.72–0.82)
	Neurological conditions	0.96(0.89–1.03)	1.04(0.85–1.28)	0.75(0.59–0.95)	0.73(0.56–0.96)
	Other deaths	0.92(0.9–0.95)	0.86(0.79–0.95)	0.84(0.78–0.91)	0.92(0.88–0.96)
Region	East Midlands	Ref	Ref	Ref	Ref
	London	1.05(1.00–1.10)	0.92(0.8–1.05)	1.07(0.95–1.21)	1.05(0.98–1.13)
	Midlands	1.00(0.96–1.04)	1.00(0.9–1.13)	1.02(0.91–1.15)	1.03(0.97–1.10)
	North-East and Yorkshire	0.93(0.89–0.98)	0.91(0.8–1.03)	1.03(0.92–1.17)	0.95(0.89–1.01)
	North-West	0.89(0.85–0.93)	0.87(0.76–0.99)	1.01(0.9–1.14)	0.96(0.9–1.03)
	South-East	0.96(0.91–1.00)	0.86(0.75–0.98)	0.94(0.83–1.07)	0.97(0.9–1.04)
	South-West	0.92(0.87–0.97)	0.91(0.79–1.04)	0.87(0.74–1.01)	0.95(0.88–1.02)

*CBD, cerebrovascular diseases; COPD, chronic obstructive pulmonary diseases; CVD, cardiovascular disease: The association is measured by proportional ratios (PRs) derived from modified Poisson regression model and 95% confidence intervals. PR > 1 indicates a higher probability of Multiple end-of-life hospitalisations, PR < 1 indicates a lower chance of Multiple end-of-life hospitalisations, PR = 1 indicates no association

The result of sensitivity analysis based on a cohort of patients in the final 30 days of life indicated that older age and residing in an urban area elevated the risk of multiple end-of-life hospital admissions among patients with diagnosed with depressive or recurrent depressive disorder, schizoaffective disorder or bipolar affective disorder and schizophrenia, schizotypal or delusional disorders (Table S5).

## Discussions

In this population-based observational study, we found that multiple end-of-life hospitalisations are prevalent among patients with mental illness, who on average experienced three hospital admissions in the last 90 days of life, with the highest rates in the London. As expected, depressive or recurrent depressive disorder was the most common recorded psychiatric diagnosis. Among patients with a diagnosis of depressive, greater socioeconomic deprivation was independently associated with multiple end-of-life hospitalisations.

Our results indicate that approximately 50% of patients with mental health conditions experienced multiple hospital end-of-life admissions. This finding align with evidence from non-end-of life studies, which have also found a similarly high rate of re-admissions among individuals with mental health illness. For example, Ronaldson and colleagues, in a systematic review and meta-analysis, found that “readmission rates within 30 days are more likely in patients with severe mental illness (pooled OR = 1.37, 95% CI 1.28–1.47, *p* < 0.001, I^2^ = 83%)” [4]. In addition, Launders and colleagues using UK primary care data showed that admission rates were twice higher in the severe mental group compared to the non-severe mental health group [28].

We found that among patients with substance use disorders, having 3 or fewer contributory causes of death was associated with multiple end-of-life hospitalisations. This finding is consistent with the high standardised mortality ratio for deaths from “all other causes” of 5.09 (95% CI 4.34, 5.94) reported in people with substance use disorder during the second quarter of 2020, the first wave of the COVID-19 pandemic [29].

Older age was associated with multiple end-of-life hospitalisations. The underlying cause of death was a relevant factor among those with depressive disorders or schizoaffective disorder or bipolar affective disorder. In other diagnostic groups, patients were less likely to have multiple end-of-life hospitalisations if the underlying cause of death was CBD, CVD, and other deaths, compared to cancer, which had similar rates of hospitalisation to COPD. Patients with COPD and depression experience more acute incidence of rehospitalisation [30]. Rates of COPD are highly prevalent in patients with severe mental illnesses [31]. COPD is an ‘ambulatory care-sensitive condition’ [32] meaning that intervention in primary or community settings can prevent unnecessary hospital admissions. If admitted however, those with a diagnosis of mental illness are more likely than the general population stay in hospital for longer (OR 1.24, 95% CI 1.12–1.37) and to be readmitted within 30-days (OR 1.51, 95% CI 1.34–1.69) although a relationship was not found with in-hospital mortality [33]. Our data highlight the importance of the provision of community-based interventions to reduce unnecessary or avoidable hospitalisation for patients with mental illness living with COPD.

In terms of place of death, the proportion of deaths occurring at home was the lowest, across all regions. This may be due to limited access to community-based palliative care, or it could perhaps be due to many people with mental illness residing in residential care facilities, which would be included in one of the ‘establishment’ categories. More precise data are therefore needed to determine whether strengthening community support and community mental health services could reduce deaths in hospitals and/or the use of acute services nearer to death [34]. Advance care planning and timely communication with patients and caregivers may increase the likelihood of home deaths [8] among individuals with mental illness.

Multiple end-of-life hospital admissions were chosen as the outcome for this study as it is an indicator of substandard palliative or end-of-life care. Also referred to as ‘burdensome transitions’, a measure of multiple hospital admissions at the end of life is often used in palliative and end of life care research as an indicator of poor care and unmet needs [1]. This measure is also used to compare end-of-life care between different settings and different patient populations [1]. Repeated hospital admissions have implications for achieving patients’ preferred place of care and death. People who had multiple admissions in the last three months of life are often not in receipt of hospice or palliative care [35]. Additionally, multiple admissions are associated with lower satisfaction of end of life care [36] and can cause significant for patients and their loved ones or carers [36].

### Study strength and limitations

To our knowledge, this is the first study using a national cohort of people with mental illness to explore factors associated with multiple end-of-life hospitalisations in England. The main strength of this study is the use of a nationally representative sample of patients with mental illness from the HES-ONS linked database. This offers better generalisability, compared to previous studies that have used limited samples from a single region or a large urban centre. Furthermore, record linkage between the HES APC and ONS enabled us to explore a range of variables, such as diagnosis of mental illness and place of death, thus giving us an insight into a wide range of factors associated with multiple end-of-life hospitalisations for each group.

Our study has several limitations common to many studies using routinely collected data. The study design is observational; therefore, we cannot make causal inferences regarding the association between end-of-life hospitalisation and sociodemographic factors. We did have access to control data representing people without diagnosis of mental illness and so cannot compare with the general population. However, from the literature the national data in England suggests that 16% of all people who died, spent 30 days or more in hospital at the end-of-life, many of which will also experience multiple hospital admissions [37].

Additionally, our study outcome was based on the number of hospital admissions at the end-of-life. Although multiple multiple hospital admissions do not always reflect poor use of health services, frequent hospitalisation during the final stages of life is often considered a marker of suboptimal palliative care. Ideally patients should be provided adequate care to ensure they are comfortable in their preferred place of care, which is unlikely to be the hospital. Previous studies have linked repeated repeated hospital admissions at the end of life to poor outcomes [38–41]. in this study, we lacked data on the reasons for multiple hospital admissions. Therefore, our findings should be cautiously interpreted because not all admissions at the end-of-life are associated with poor outcomes.

 We used aggregate or area-level socioeconomic status (IMD) as a proxy for individual-level socioeconomic position. This introduces a risk of ecological fallacy, as aggregate data may not reflect individual socioeconomic status. Similarly, the number of contributory causes of death was used as a proxy for comorbidity. Although this approach gives an indication of disease burden, more robust measures such as, the Charlson index or Elixhauser should be used in future research.

Our analysis may also have overestimated the size of the association between patients’ sociodemographic variables and multiple end-of-life hospitalisation, as we were unable to account for differences in community service or accessibility to primary care provisions. For example, access to primary care could influence frequency of hospital admissions. Further research should account for the role of alternative care provisions (e.g., informal care, primary care), on hospitalisations. This research should also be replicated in post-COVID-19 data and in other countries.

Finally, categorisations of place of death in the ONS data was limited to only four groups: Home, NHS communal establishments, non-NHS communal establishments, and elsewhere and other places (e.g., hostels, prisons, detention centres, public spaces). Due to these limited categories, we were unable to ascertain the proportion of deaths in acute hospitals. It is important that future research use more refined categorisation of establish the proportion of deaths in acute hospitals using appropriate routine data.

## Conclusion

Multiple end-of-life hospitalisations are high among people with mental illness. As with the general population, these hospitalisation burden to the patient and to their families and carers, as well as a cost to the health care system, and it is likely that many are avoidable. Most health care systems in the UK and other countries in the Global North have a clear dichotomy between physical and psychiatric medicine [42]. This division increases the risk of care fragmentation and potentially unnecessary hospital admissions. Greater integration between physical and secondary or mental health has the potential to reduce multiple hospitalisations at the end-of-life among individuals with mental illness [43].

Our findings suggest that more targeted approaches such as strengthening primary and community care services particularly in urban areas and regions with high levels of socioeconomic deprivation, could help reduce multiple end-of-life hospital admissions. Developing tailored services for older and those with cancer or COPD may help to minmise unnecessary end-of-life hospitalisations among individuals with mental illness.

## Electronic supplementary material

Below is the link to the electronic supplementary material.

Supplementary Material 1

## Data Availability

The data that support the findings of this study are available from the NHS Digital, but restrictions apply to the availability of these data, which were used under license for the current study, and so are not publicly available.
