# Supplementary material for: Geographic variations in end-of-life hospitalisations for patients with mental illness: a population-based observational study in England, UK
Source: Discov Ment Health. 2025 Oct 6;5(1):147. doi: 10.1007/s44192-025-00252-z (PMC12500490; doi:10.1007/s44192-025-00252-z)
Supplement: Supplementary file 1 — Supplementary Material 1 [file 44192_2025_252_MOESM1_ESM.docx]

**Table S1: Regional variations in end-of-life hospitalisation for patients with a diagnosis of Depressive episode or Recurrent depressive disorder**

|  | level | Overall | East-of-England | London | Midlands | Northeast &Yorkshire | North-West | South-East | South-West |
| --- | --- | --- | --- | --- | --- | --- | --- | --- | --- |
| n |  | 15130 | 1902 | 1762 | 3244 | 2545 | 2012 | 2372 | 1293 |
| Gender (%) | Female | 8923 (59.0) | 1133 (59.6) | 1008 (57.2) | 1918 (59.1) | 1515 (59.5) | 1185 (58.9) | 1399 (59.0) | 765 (59.2) |
|  | Male | 6207 (41.0) | 769 (40.4) | 754 (42.8) | 1326 (40.9) | 1030 (40.5) | 827 (41.1) | 973 (41.0) | 528 (40.8) |
| Age (mean (SD)) |  | 77.18 (13.54) | 78.43 (13.11) | 77.34 (13.74) | 77.02 (13.56) | 76.45 (13.17) | 75.27 (14.27) | 78.58 (13.16) | 77.32 (13.64) |
| Age group (%) | 18-54 | 1044 ( 6.9) | 113 ( 5.9) | 121 ( 6.9) | 227 ( 7.0) | 171 ( 6.7) | 193 ( 9.6) | 127 ( 5.4) | 92 ( 7.1) |
|  | 55-64 | 1544 (10.2) | 170 ( 8.9) | 170 ( 9.6) | 342 (10.5) | 274 (10.8) | 246 (12.2) | 214 ( 9.0) | 128 ( 9.9) |
|  | 65-74 | 2915 (19.3) | 336 (17.7) | 346 (19.6) | 630 (19.4) | 545 (21.4) | 404 (20.1) | 435 (18.3) | 219 (16.9) |
|  | 75-84 | 4327 (28.6) | 541 (28.4) | 483 (27.4) | 939 (28.9) | 737 (29.0) | 559 (27.8) | 664 (28.0) | 404 (31.2) |
|  | 85+ | 5300 (35.0) | 742 (39.0) | 642 (36.4) | 1106 (34.1) | 818 (32.1) | 610 (30.3) | 932 (39.3) | 450 (34.8) |
| Underlying cause of death (%) | Cancers | 4112 (27.2) | 508 (26.7) | 509 (28.9) | 844 (26.0) | 702 (27.6) | 534 (26.5) | 633 (26.7) | 382 (29.5) |
|  | CBDs | 886 ( 5.9) | 123 ( 6.5) | 99 ( 5.6) | 197 ( 6.1) | 154 ( 6.1) | 110 ( 5.5) | 131 ( 5.5) | 72 ( 5.6) |
|  | COPDs | 1295 ( 8.6) | 132 ( 6.9) | 128 ( 7.3) | 275 ( 8.5) | 253 ( 9.9) | 192 ( 9.5) | 218 ( 9.2) | 97 ( 7.5) |
|  | CVDs | 1776 (11.7) | 222 (11.7) | 214 (12.1) | 381 (11.7) | 313 (12.3) | 198 ( 9.8) | 303 (12.8) | 145 (11.2) |
|  | Neurological conditions | 371 ( 2.5) | 52 ( 2.7) | 42 ( 2.4) | 82 ( 2.5) | 61 ( 2.4) | 39 ( 1.9) | 69 ( 2.9) | 26 ( 2.0) |
|  | Other Deaths | 6690 (44.2) | 865 (45.5) | 770 (43.7) | 1465 (45.2) | 1062 (41.7) | 939 (46.7) | 1018 (42.9) | 571 (44.2) |
| Year of death (%) | 2018 | 10817 (71.5) | 1361 (71.6) | 1259 (71.5) | 2321 (71.5) | 1842 (72.4) | 1456 (72.4) | 1626 (68.5) | 952 (73.6) |
|  | 2019 | 4313 (28.5) | 541 (28.4) | 503 (28.5) | 923 (28.5) | 703 (27.6) | 556 (27.6) | 746 (31.5) | 341 (26.4) |
| Place of death (%) | Elsewhere/Other Places | 1355 ( 9.0) | 192 (10.1) | 164 ( 9.3) | 272 ( 8.4) | 236 ( 9.3) | 188 ( 9.3) | 180 ( 7.6) | 123 ( 9.5) |
|  | Home | 880 ( 5.8) | 119 ( 6.3) | 107 ( 6.1) | 172 ( 5.3) | 160 ( 6.3) | 118 ( 5.9) | 132 ( 5.6) | 72 ( 5.6) |
|  | NHS Establishments | 9245 (61.1) | 1097 (57.7) | 1134 (64.4) | 2047 (63.1) | 1534 (60.3) | 1247 (62.0) | 1423 (60.0) | 763 (59.0) |
|  | Non-NHS Establishments | 3650 (24.1) | 494 (26.0) | 357 (20.3) | 753 (23.2) | 615 (24.2) | 459 (22.8) | 637 (26.9) | 335 (25.9) |
| Ethnicity (%) | Asian | 296 ( 2.0) | 22 ( 1.2) | 121 ( 6.9) | 74 ( 2.3) | 24 ( 0.9) | 27 ( 1.3) | 26 ( 1.1) | < 10* |
|  | Black/Mixed/Others | 284 ( 1.9) | 18 ( 0.9) | 152 ( 8.6) | 40 ( 1.2) | 13 ( 0.5) | 24 ( 1.2) | 25 ( 1.1) | 12 ( 0.9) |
|  | British(White) | 12945 (85.6) | 1646 (86.5) | 1137 (64.5) | 2841 (87.6) | 2337 (91.8) | 1834 (91.2) | 1998 (84.2) | 1152 (89.1) |
|  | Irish(White) | 151 ( 1.0) | 11 ( 0.6) | 63 ( 3.6) | 34 ( 1.0) | < 10* | 17 ( 0.8) | 17 ( 0.7) | < 10* |
|  | Not Stated / Not known | 1050 ( 6.9) | 160 ( 8.4) | 147 ( 8.3) | 198 ( 6.1) | < 10* | 88 ( 4.4) | 211 ( 8.9) | 103 ( 8.0) |
|  | Others(White) | 404 ( 2.7) | 45 ( 2.4) | 142 ( 8.1) | 57 ( 1.8) | 25 ( 1.0) | 22 ( 1.1) | 95 ( 4.0) | 18 ( 1.4) |
| Contributory cause of death (%) | 0 | 2855 (18.9) | 328 (17.2) | 300 (17.0) | 568 (17.5) | 518 (20.4) | 446 (22.2) | 435 (18.3) | 260 (20.1) |
|  | 1 | 3340 (22.1) | 436 (22.9) | 361 (20.5) | 668 (20.6) | 546 (21.5) | 511 (25.4) | 490 (20.7) | 328 (25.4) |
|  | 2 | 3264 (21.6) | 421 (22.1) | 374 (21.2) | 734 (22.6) | 537 (21.1) | 413 (20.5) | 486 (20.5) | 299 (23.1) |
|  | 3 | 2369 (15.7) | 319 (16.8) | 292 (16.6) | 533 (16.4) | 409 (16.1) | 255 (12.7) | 380 (16.0) | 181 (14.0) |
|  | 4+ | 3302 (21.8) | 398 (20.9) | 435 (24.7) | 741 (22.8) | 535 (21.0) | 387 (19.2) | 581 (24.5) | 225 (17.4) |
| Settlement (%) | Rural | 1367 ( 9.0) | 264 (13.9) | < 10* | 342 (10.5) | 182 ( 7.2) | 69 ( 3.4) | 294 (12.4) | 212 (16.4) |
|  | Urban | 13763 (91.0) | 1638 (86.1) | < 10* | 2902 (89.5) | 2363 (92.8) | 1943 (96.6) | 2078 (87.6) | 1081 (83.6) |
| Socioeconomic status (%) | 1 | 3607 (23.8) | 232 (12.2) | 303 (17.2) | 822 (25.3) | 968 (38.0) | 859 (42.7) | 246 (10.4) | 177 (13.7) |
|  | 2 | 3207 (21.2) | 396 (20.8) | 526 (29.9) | 671 (20.7) | 526 (20.7) | 404 (20.1) | 404 (17.0) | 280 (21.7) |
|  | 3 | 3079 (20.4) | 493 (25.9) | 391 (22.2) | 681 (21.0) | 415 (16.3) | 294 (14.6) | 481 (20.3) | 324 (25.1) |
|  | 4 | 2764 (18.3) | 426 (22.4) | 310 (17.6) | 574 (17.7) | 365 (14.3) | 256 (12.7) | 552 (23.3) | 281 (21.7) |
|  | 5 | 2473 (16.3) | 355 (18.7) | 232 (13.2) | 496 (15.3) | 271 (10.6) | 199 ( 9.9) | 689 (29.0) | 231 (17.9) |
| Number.of.admissions.last.90.days (median [IQR]) |  | 3.00 [2.00, 4.00] | 3.00 [2.00, 4.00] | 3.00 [2.00, 5.00] | 3.00 [2.00, 4.00] | 3.00 [2.00, 4.00] | 3.00 [2.00, 4.00] | 3.00 [2.00, 4.00] | 3.00 [2.00, 4.00] |

*These values are suppressed to avoid secondary disclosure. CBD—Cerebrovascular diseases; COPD—Chronic obstructive pulmonary diseases; CVD—cardiovascular disease:

**Table S2: Regional variations in multiple end-of-life hospitalisation for patients with a diagnosis of schizoaffective disorder or Bipolar affective disorder**

|  | level | Overall | East-of-England | London | Midlands | Northeast Yorkshire | North-West | South-East | South-West |
| --- | --- | --- | --- | --- | --- | --- | --- | --- | --- |
| n |  | 1259 | 185 | 168 | 227 | 206 | 168 | 172 | 133 |
| Gender (%) | Female | 781 (62.0) | 116 (62.7) | 106 ( 63.1) | 133 (58.6) | 123 (59.7) | 116 (69.0) | 100 (58.1) | 87 (65.4) |
|  | Male | 478 (38.0) | 69 (37.3) | 62 ( 36.9) | 94 (41.4) | 83 (40.3) | 52 (31.0) | 72 (41.9) | 46 (34.6) |
| Age (mean (SD)) |  | 72.93 (13.00) | 74.42 (12.01) | 73.05 (14.52) | 73.44 (12.35) | 72.50 (13.05) | 70.49 (12.55) | 74.81 (13.19) | 71.21 (13.13) |
| Age group (%) | 18-54 | 118 ( 9.4) | 13 ( 7.0) | 20 ( 11.9) | 19 ( 8.4) | 18 ( 8.7) | 20 (11.9) | 15 ( 8.7) | 13 ( 9.8) |
|  | 55-64 | 171 (13.6) | 22 (11.9) | 21 ( 12.5) | 28 (12.3) | 33 (16.0) | 30 (17.9) | 20 (11.6) | 17 (12.8) |
|  | 65-74 | 317 (25.2) | 45 (24.3) | 35 ( 20.8) | 65 (28.6) | 52 (25.2) | 50 (29.8) | 32 (18.6) | 38 (28.6) |
|  | 75-84 | 416 (33.0) | 64 (34.6) | 57 ( 33.9) | 69 (30.4) | 65 (31.6) | 45 (26.8) | 61 (35.5) | 55 (41.4) |
|  | 85+ | 237 (18.8) | 41 (22.2) | 35 ( 20.8) | 46 (20.3) | 38 (18.4) | 23 (13.7) | 44 (25.6) | < 10* |
| Underlying cause of death (%) | Cancers | 291 (23.1) | 49 (26.5) | 36 ( 21.4) | 53 (23.3) | 42 (20.4) | 40 (23.8) | 38 (22.1) | 33 (24.8) |
|  | CBDs | 61 ( 4.8) | < 10* | 13 ( 7.7) | < 10* | < 10* | < 10* | < 10* | < 10* |
|  | COPDs | 93 ( 7.4) | 12 ( 6.5) | 14 ( 8.3) | < 10* | 20 ( 9.7) | 20 (11.9) | 12 ( 7.0) | 5 ( 3.8) |
|  | CVDs | 163 (12.9) | 20 (10.8) | 22 ( 13.1) | 32 (14.1) | 25 (12.1) | 16 ( 9.5) | 27 (15.7) | 21 (15.8) |
|  | Neurological conditions | 32 ( 2.5) | < 10* | < 10* | < 10* | < 10* | < 10* | < 10* | < 10* |
|  | Other Deaths | 619 (49.2) | 89 (48.1) | 77 ( 45.8) | 120 (52.9) | 106 (51.5) | 82 (48.8) | 79 (45.9) | 66 (49.6) |
| Year of Death (%) | 2018 | 899 (71.4) | 131 (70.8) | 124 ( 73.8) | 160 (70.5) | 148 (71.8) | 121 (72.0) | 123 (71.5) | 92 (69.2) |
|  | 2019 | 360 (28.6) | 54 (29.2) | 44 ( 26.2) | 67 (29.5) | 58 (28.2) | 47 (28.0) | 49 (28.5) | 41 (30.8) |
| Place of death (%) | Elsewhere/Other Places | 119 ( 9.5) | 22 (11.9) | 11 ( 6.5) | 22 ( 9.7) | 14 ( 6.8) | 19 (11.3) | 18 (10.5) | 13 ( 9.8) |
|  | Home | 58 ( 4.6) | < 10* | < 10* | < 10* | < 10* | < 10* | 11 ( 6.4) | < 10* |
|  | NHS Establishments | 763 (60.6) | 105 (56.8) | 104 ( 61.9) | 146 (64.3) | 119 (57.8) | 106 (63.1) | 95 (55.2) | 88 (66.2) |
|  | Non-NHS Establishments | 319 (25.3) | 50 (27.0) | 44 ( 26.2) | 50 (22.0) | 63 (30.6) | 37 (22.0) | 48 (27.9) | 27 (20.3) |
| Ethnicity (%) | Asian | 34 ( 2.7) | < 10* | 16 ( 9.5) | < 10* | < 10* | < 10* | < 10* | < 10* |
|  | Black/Mixed/Others | 41 ( 3.3) | < 10* | 17 ( 10.1) | < 10* | < 10* | < 10* | < 10* | < 10* |
|  | British(White) | 1053 (83.6) | 163 (88.1) | 106 ( 63.1) | 190 (83.7) | 191 (92.7) | 149 (88.7) | 138 (80.2) | 116 (87.2) |
|  | Irish(White) | 17 ( 1.4) | < 10* | < 10* | < 10* | < 10* | < 10* | < 10* | < 10* |
|  | Not Stated/ Not known | 81 ( 6.4) | 12 ( 6.5) | 15 ( 8.9) | 14 ( 6.2) | < 10* | < 10* | 17 ( 9.9) | < 10* |
|  | Others(White) | 33 ( 2.6) | < 10* | < 10* | < 10* | < 10* | < 10* | < 10* | < 10* |
| Contributory causes of death (%) | 0 | 213 (16.9) | 32 (17.3) | 19 ( 11.3) | 40 (17.6) | 40 (19.4) | 37 (22.0) | 26 (15.1) | 19 (14.3) |
|  | 1 | 283 (22.5) | 42 (22.7) | 41 ( 24.4) | 51 (22.5) | 41 (19.9) | 31 (18.5) | 41 (23.8) | 36 (27.1) |
|  | 2 | 265 (21.0) | 34 (18.4) | 37 ( 22.0) | 42 (18.5) | 46 (22.3) | 38 (22.6) | 42 (24.4) | 26 (19.5) |
|  | 3 | 198 (15.7) | 27 (14.6) | 25 ( 14.9) | 36 (15.9) | 35 (17.0) | 28 (16.7) | 26 (15.1) | 21 (15.8) |
|  | 4+ | 300 (23.8) | 50 (27.0) | 46 ( 27.4) | 58 (25.6) | 44 (21.4) | 34 (20.2) | 37 (21.5) | 31 (23.3) |
| Settlement (%) | Rural | 94 ( 7.5) | 25 (13.5) | < 10* | < 10* | < 10* | < 10* | 19 (11.0) | 19 (14.3) |
|  | Urban | 1165 (92.5) | 160 (86.5) | < 10* | < 10* | < 10* | < 10* | 153 (89.0) | 114 (85.7) |
| Socioeconomic status (%) | 1 | 306 (24.3) | 27 (14.6) | 23 ( 13.7) | 70 (30.8) | 81 (39.3) | 72 (42.9) | 14 ( 8.1) | 19 (14.3) |
|  | 2 | 301 (23.9) | 39 (21.1) | 62 ( 36.9) | 48 (21.1) | 40 (19.4) | 42 (25.0) | 35 (20.3) | 35 (26.3) |
|  | 3 | 255 (20.3) | 49 (26.5) | 31 ( 18.5) | 44 (19.4) | 33 (16.0) | 25 (14.9) | 37 (21.5) | 36 (27.1) |
|  | 4 | 223 (17.7) | 36 (19.5) | 40 ( 23.8) | 34 (15.0) | 32 (15.5) | 17 (10.1) | 38 (22.1) | 26 (19.5) |
|  | 5 | 174 (13.8) | 34 (18.4) | 12 ( 7.1) | 31 (13.7) | 20 ( 9.7) | 12 ( 7.1) | 48 (27.9) | 17 (12.8) |
| Number.of.admissions.last.90.days (median [IQR]) |  | 3.00 [2.00, 5.00] | 3.00 [2.00, 5.00] | 3.00 [2.00, 5.00] | 3.00 [2.00, 5.00] | 3.00 [2.00, 4.00] | 3.00 [2.00, 5.00] | 3.00 [2.00, 5.00] | 4.00 [2.00, 5.00] |

*These values are suppressed to avoid secondary disclosure. CBD—Cerebrovascular diseases; COPD—Chronic obstructive pulmonary diseases; CVD—cardiovascular disease:

**Table S3: Regional variations in end-of-life hospitalisation for patients with a diagnosis of Schizotypal, Delusional disorders & Schizophrenia**

|  | level | Overall | East-of-England | London | Midlands | North-East & Yorkshire | North-West | South-East | South-West |
| --- | --- | --- | --- | --- | --- | --- | --- | --- | --- |
| n |  | 2072 | 205 | 400 | 387 | 357 | 330 | 248 | 145 |
| Gender (%) | Female | 1052 (50.8) | 120 (58.5) | 192 ( 48.0) | 190 (49.1) | 187 (52.4) | 169 (51.2) | 123 (49.6) | 71 (49.0) |
|  | Male | 1020 (49.2) | 85 (41.5) | 208 ( 52.0) | 197 (50.9) | 170 (47.6) | 161 (48.8) | 125 (50.4) | 74 (51.0) |
| Age (mean (SD)) |  | 72.98 (13.78) | 74.39 (14.61) | 72.14 (13.41) | 73.18 (13.95) | 73.66 (13.77) | 71.91 (13.56) | 72.33 (13.67) | 74.64 (13.63) |
| Age group (%) | 18-54 | 222 (10.7) | 20 ( 9.8) | 49 ( 12.2) | 46 (11.9) | 38 (10.6) | 35 (10.6) | 23 ( 9.3) | 11 ( 7.6) |
|  | 55-64 | 293 (14.1) | 21 (10.2) | 54 ( 13.5) | 49 (12.7) | 48 (13.4) | 58 (17.6) | 42 (16.9) | 21 (14.5) |
|  | 65-74 | 530 (25.6) | 53 (25.9) | 99 ( 24.8) | 97 (25.1) | 83 (23.2) | 84 (25.5) | 75 (30.2) | 39 (26.9) |
|  | 75-84 | 587 (28.3) | 60 (29.3) | 136 ( 34.0) | 108 (27.9) | 104 (29.1) | 93 (28.2) | 55 (22.2) | 31 (21.4) |
|  | 85+ | 440 (21.2) | 51 (24.9) | 62 ( 15.5) | 87 (22.5) | 84 (23.5) | 60 (18.2) | 53 (21.4) | 43 (29.7) |
| Underlying cause of death (%) | Cancers | 500 (24.1) | 51 (24.9) | 94 ( 23.5) | 95 (24.5) | 73 (20.4) | 83 (25.2) | 65 (26.2) | 39 (26.9) |
|  | CBDs | 119 ( 5.7) | < 10* | 21 ( 5.2) | 21 ( 5.4) | 21 ( 5.9) | 16 ( 4.8) | 20 ( 8.1) | < 10* |
|  | COPDs | 180 ( 8.7) | < 10* | 28 ( 7.0) | 37 ( 9.6) | 40 (11.2) | 32 ( 9.7) | 14 ( 5.6) | 15 (10.3) |
|  | CVDs | 233 (11.2) | 24 (11.7) | 54 ( 13.5) | 41 (10.6) | 38 (10.6) | 31 ( 9.4) | 27 (10.9) | 18 (12.4) |
|  | Neurological conditions | 37 ( 1.8) | < 10* | < 10* | < 10* | < 10* | < 10* | < 10* | < 10* |
|  | Other Deaths | 1003 (48.4) | 104 (50.7) | 197 ( 49.2) | 183 (47.3) | 175 (49.0) | 164 (49.7) | 118 (47.6) | 62 (42.8) |
| Year of death (%) | 2018 | 1469 (70.9) | 147 (71.7) | 287 ( 71.8) | 253 (65.4) | 252 (70.6) | 248 (75.2) | 183 (73.8) | 99 (68.3) |
|  | 2019 | 603 (29.1) | 58 (28.3) | 113 ( 28.2) | 134 (34.6) | 105 (29.4) | 82 (24.8) | 65 (26.2) | 46 (31.7) |
| Place of death (%) | Elsewhere/Other Places | 189 ( 9.1) | 20 ( 9.8) | 45 ( 11.2) | 28 ( 7.2) | 32 ( 9.0) | 32 ( 9.7) | 19 ( 7.7) | 13 ( 9.0) |
|  | Home | 96 ( 4.6) | 5 ( 2.4) | 27 ( 6.8) | 14 ( 3.6) | 17 ( 4.8) | 16 ( 4.8) | < 10* | < 10* |
|  | NHS Establishments | 1290 (62.3) | 125 (61.0) | 255 ( 63.7) | 251 (64.9) | 215 (60.2) | 213 (64.5) | 149 (60.1) | 82 (56.6) |
|  | Non-NHS Establishments | 497 (24.0) | 55 (26.8) | 73 ( 18.2) | 94 (24.3) | 93 (26.1) | 69 (20.9) | 71 (28.6) | 42 (29.0) |
| Ethnicity (%) | Asian | 103 ( 5.0) | < 10* | 42 ( 10.5) | 33 ( 8.5) | < 10* | < 10* | < 10* | < 10* |
|  | Black/Mixed/Others | 146 ( 7.0) | < 10* | 93 ( 23.2) | 21 ( 5.4) | < 10* | < 10* | < 10* | < 10* |
|  | British(White) | 1577 (76.1) | 163 (79.5) | 181 ( 45.2) | 300 (77.5) | 317 (88.8) | 285 (86.4) | 208 (83.9) | 123 (84.8) |
|  | Irish(White) | 28 ( 1.4) | < 10* | 12 ( 3.0) | < 10* | < 10* | < 10* | < 10* | < 10* |
|  | Not Stated/Not known | 155 ( 7.5) | 21 (10.2) | 47 ( 11.8) | 26 ( 6.7) | 16 ( 4.5) | 15 ( 4.5) | 19 ( 7.7) | 11 ( 7.6) |
|  | Others(White) | 63 ( 3.0) | < 10* | 25 ( 6.2) | < 10* | < 10* | < 10* | < 10* | < 10* |
| Contributory causes of death (%) | 0 | 333 (16.1) | 24 (11.7) | 58 ( 14.5) | 67 (17.3) | 58 (16.2) | 60 (18.2) | 33 (13.3) | 33 (22.8) |
|  | 1 | 471 (22.7) | 41 (20.0) | 84 ( 21.0) | 83 (21.4) | 86 (24.1) | 86 (26.1) | 48 (19.4) | 43 (29.7) |
|  | 2 | 455 (22.0) | 58 (28.3) | 82 ( 20.5) | 80 (20.7) | 83 (23.2) | 65 (19.7) | 58 (23.4) | 29 (20.0) |
|  | 3 | 326 (15.7) | 42 (20.5) | 62 ( 15.5) | 62 (16.0) | 59 (16.5) | 49 (14.8) | 37 (14.9) | 15 (10.3) |
|  | 4+ | 487 (23.5) | 40 (19.5) | 114 ( 28.5) | 95 (24.5) | 71 (19.9) | 70 (21.2) | 72 (29.0) | 25 (17.2) |
| Settlement (%) | Rural | 121 ( 5.8) | 24 (11.7) | < 10* | 33 ( 8.5) | 17 ( 4.8) | < 10* | 23 ( 9.3) | 16 (11.0) |
|  | Urban | 1951 (94.2) | 181 (88.3) | 400 (100.0) | 354 (91.5) | 340 (95.2) | 322 (97.6) | 225 (90.7) | 129 (89.0) |
| Socioeconomic status (%) | 1 | 669 (32.3) | 32 (15.6) | 102 ( 25.5) | 135 (34.9) | 173 (48.5) | 169 (51.2) | 36 (14.5) | 22 (15.2) |
|  | 2 | 501 (24.2) | 47 (22.9) | 136 ( 34.0) | 100 (25.8) | 66 (18.5) | 61 (18.5) | 49 (19.8) | 42 (29.0) |
|  | 3 | 384 (18.5) | 51 (24.9) | 76 ( 19.0) | 69 (17.8) | 59 (16.5) | 41 (12.4) | 55 (22.2) | 33 (22.8) |
|  | 4 | 295 (14.2) | 45 (22.0) | 52 ( 13.0) | 44 (11.4) | 43 (12.0) | 35 (10.6) | 49 (19.8) | 27 (18.6) |
|  | 5 | 223 (10.8) | 30 (14.6) | 34 ( 8.5) | 39 (10.1) | 16 ( 4.5) | 24 ( 7.3) | 59 (23.8) | 21 (14.5) |
| Number.of.admissions.last.90.days (median [IQR]) |  | 3.00 [2.00, 5.00] | 3.00 [2.00, 4.00] | 3.00 [2.00, 5.00] | 3.00 [2.00, 5.00] | 3.00 [2.00, 4.00] | 3.00 [2.00, 4.00] | 3.00 [2.00, 5.00] | 3.00 [2.00, 5.00] |

*These values are suppressed to avoid secondary disclosure. CBD—Cerebrovascular diseases; COPD—Chronic obstructive pulmonary diseases; CVD—cardiovascular disease:

**Table S4: Regional variations in end-of-life hospitalisation for patients with a diagnosis of substance use disorders**

|  | level | Overall | East-of-England | London | Midlands | North-East  & Yorkshire | | North-West | South-East | South-West |
| --- | --- | --- | --- | --- | --- | --- | --- | --- | --- | --- |
| n |  | 6543 | 617 | 755 | 1310 | 1229 | 1272 | | 788 | 572 |
| Gender (%) | Female | 2979 (45.5) | 270 (43.8) | 313 ( 41.5) | 589 (45.0) | 581 (47.3) | 604 (47.5) | | 365 (46.3) | 257 (44.9) |
|  | Male | 3564 (54.5) | 347 (56.2) | 442 ( 58.5) | 721 (55.0) | 648 (52.7) | 668 (52.5) | | 423 (53.7) | 315 (55.1) |
| Age (mean (SD)) |  | 63.21 (13.55) | 63.94 (13.69) | 64.26 (13.53) | 62.79 (13.92) | 62.85 (13.87) | 62.31 (12.94) | | 64.65 (13.41) | 62.76 (13.18) |
| Age group (%) | 18-54 | 1774 (27.1) | 158 (25.6) | 192 ( 25.4) | 361 (27.6) | 348 (28.3) | 362 (28.5) | | 194 (24.6) | 159 (27.8) |
|  | 55-64 | 1649 (25.2) | 151 (24.5) | 175 ( 23.2) | 330 (25.2) | 308 (25.1) | 351 (27.6) | | 185 (23.5) | 149 (26.0) |
|  | 65-74 | 1705 (26.1) | 166 (26.9) | 212 ( 28.1) | 344 (26.3) | 303 (24.7) | 318 (25.0) | | 214 (27.2) | 148 (25.9) |
|  | 75-84 | 1067 (16.3) | 106 (17.2) | 133 ( 17.6) | 213 (16.3) | 206 (16.8) | 186 (14.6) | | 139 (17.6) | 84 (14.7) |
|  | 85+ | 348 ( 5.3) | 36 ( 5.8) | 43 ( 5.7) | 62 ( 4.7) | 64 ( 5.2) | 55 ( 4.3) | | 56 ( 7.1) | 32 ( 5.6) |
| Underlying causes of death (%) | Cancers | 1711 (26.2) | 177 (28.7) | 215 ( 28.5) | 328 (25.0) | 305 (24.8) | 335 (26.3) | | 207 (26.3) | 144 (25.2) |
|  | CBDs | 187 ( 2.9) | 16 ( 2.6) | 21 ( 2.8) | 40 ( 3.1) | 32 ( 2.6) | 39 ( 3.1) | | 24 ( 3.0) | 15 ( 2.6) |
|  | COPDs | 888 (13.6) | 86 (13.9) | 122 ( 16.2) | 159 (12.1) | 181 (14.7) | 155 (12.2) | | 108 (13.7) | 77 (13.5) |
|  | CVDs | 717 (11.0) | 74 (12.0) | 74 ( 9.8) | 138 (10.5) | 133 (10.8) | 148 (11.6) | | 86 (10.9) | 64 (11.2) |
|  | Neurological conditions | 29 ( 0.4) | < 10* | < 10* | < 10* | < 10* | < 10* | | < 10* | < 10* |
|  | Other Deaths | 3011 (46.0) | 262 (42.5) | 319 ( 42.3) | 636 (48.5) | 574 (46.7) | 589 (46.3) | | 361 (45.8) | 270 (47.2) |
| Year of Death (%) | 2018 | 4673 (71.4) | 432 (70.0) | 547 ( 72.5) | 945 (72.1) | 864 (70.3) | 912 (71.7) | | 548 (69.5) | 425 (74.3) |
|  | 2019 | 1870 (28.6) | 185 (30.0) | 208 ( 27.5) | 365 (27.9) | 365 (29.7) | 360 (28.3) | | 240 (30.5) | 147 (25.7) |
| Place of death (%) | Elsewhere/Other Places | 835 (12.8) | 88 (14.3) | 93 ( 12.3) | 173 (13.2) | 147 (12.0) | 152 (11.9) | | 90 (11.4) | 92 (16.1) |
|  | Home | 579 ( 8.8) | 61 ( 9.9) | 53 ( 7.0) | 112 ( 8.5) | 121 ( 9.8) | 114 ( 9.0) | | 71 ( 9.0) | 47 ( 8.2) |
|  | NHS Establishments | 4368 (66.8) | 374 (60.6) | 515 ( 68.2) | 883 (67.4) | 820 (66.7) | 875 (68.8) | | 539 (68.4) | 362 (63.3) |
|  | Non-NHS Establishments | 761 (11.6) | 94 (15.2) | 94 ( 12.5) | 142 (10.8) | 141 (11.5) | 131 (10.3) | | 88 (11.2) | 71 (12.4) |
| Ethnicity (%) | Asian | 98 ( 1.5) | < 10* | 32 ( 4.2) | 39 ( 3.0) | 12 ( 1.0) | < 10* | | < 10* | < 10* |
|  | Black/Mixed/Others | 125 ( 1.9) | < 10* | 62 ( 8.2) | 25 ( 1.9) | < 10* | < 10* | | < 10* | < 10* |
|  | British(White) | 5632 (86.1) | 526 (85.3) | 486 ( 64.4) | 1124 (85.8) | 1132 (92.1) | 1181 (92.8) | | 684 (86.8) | 499 (87.2) |
|  | Irish(White) | 89 ( 1.4) | 14 ( 2.3) | 28 ( 3.7) | 15 ( 1.1) | < 10* | 10 ( 0.8) | | 8 ( 1.0) | 5 ( 0.9) |
|  | Not Stated/Not known | 430 ( 6.6) | 45 ( 7.3) | 83 ( 11.0) | 90 ( 6.9) | 50 ( 4.1) | 53 ( 4.2) | | 60 ( 7.6) | 49 ( 8.6) |
|  | Others(White) | 169 ( 2.6) | 20 ( 3.2) | 64 ( 8.5) | 17 ( 1.3) | 14 ( 1.1) | 12 ( 0.9) | | 28 ( 3.6) | 14 ( 2.4) |
| Contributory causes of death (%) | 0 | 1197 (18.3) | 111 (18.0) | 124 ( 16.4) | 241 (18.4) | 256 (20.8) | 239 (18.8) | | 114 (14.5) | 112 (19.6) |
|  | 1 | 1463 (22.4) | 138 (22.4) | 148 ( 19.6) | 293 (22.4) | 245 (19.9) | 331 (26.0) | | 160 (20.3) | 148 (25.9) |
|  | 2 | 1407 (21.5) | 118 (19.1) | 170 ( 22.5) | 295 (22.5) | 278 (22.6) | 260 (20.4) | | 165 (20.9) | 121 (21.2) |
|  | 3 | 1027 (15.7) | 95 (15.4) | 139 ( 18.4) | 196 (15.0) | 198 (16.1) | 190 (14.9) | | 129 (16.4) | 80 (14.0) |
|  | 4+ | 1449 (22.1) | 155 (25.1) | 174 ( 23.0) | 285 (21.8) | 252 (20.5) | 252 (19.8) | | 220 (27.9) | 111 (19.4) |
| Settlement (%) | Rural | 348 ( 5.3) | 58 ( 9.4) | < 10* | 87 ( 6.6) | 51 ( 4.1) | 24 ( 1.9) | | 71 ( 9.0) | 57 (10.0) |
|  | Urban | 6195 (94.7) | 559 (90.6) | 755 (100.0) | 1223 (93.4) | 1178 (95.9) | 1248 (98.1) | | 717 (91.0) | 515 (90.0) |
| Socioeconomic status (%) | 1 | 2451 (37.5) | 116 (18.8) | 183 ( 24.2) | 516 (39.4) | 603 (49.1) | 758 (59.6) | | 140 (17.8) | 135 (23.6) |
|  | 2 | 1551 (23.7) | 146 (23.7) | 292 ( 38.7) | 280 (21.4) | 255 (20.7) | 211 (16.6) | | 218 (27.7) | 149 (26.0) |
|  | 3 | 1095 (16.7) | 163 (26.4) | 141 ( 18.7) | 210 (16.0) | 189 (15.4) | 126 ( 9.9) | | 147 (18.7) | 119 (20.8) |
|  | 4 | 831 (12.7) | 116 (18.8) | 103 ( 13.6) | 166 (12.7) | 99 ( 8.1) | 105 ( 8.3) | | 146 (18.5) | 96 (16.8) |
|  | 5 | 615 ( 9.4) | 76 (12.3) | 36 ( 4.8) | 138 (10.5) | 83 ( 6.8) | 72 ( 5.7) | | 137 (17.4) | 73 (12.8) |
| Number.of.admissions.last.90.days (median [IQR]) |  | 3.00 [2.00, 5.00] | 3.00 [2.00, 4.00] | 3.00 [2.00, 5.00] | 3.00 [2.00, 5.00] | 3.00 [2.00, 5.00] | 3.00 [2.00, 5.00] | | 3.00 [2.00, 5.00] | 3.00 [2.00, 5.00] |

*These values are suppressed to avoid secondary disclosure. CBD—Cerebrovascular diseases; COPD—Chronic obstructive pulmonary diseases; CVD—cardiovascular disease:

**Table S5: Factors associated with Multiple EoL for patients in the last 30 days of life by diagnosis of mental illness (sensitivity analysis)**

| Variables | Levels | Depressive episode or Recurrent depressive disorder | Schizoaffective disorder or Bipolar affective disorder | Schizotypal/Delusional disorders/Schizophrenia | Substance use disorders |  |
| --- | --- | --- | --- | --- | --- | --- |
|  |  |  |  |  |  |  |
|  |  | PR (95% CI.) | PR (95% CI.) | PR (95% CI.) | PR (95% CI.) |  |
| Gender | Female | Ref | Ref | Ref | Ref |  |
|  | Male | 1.00(0.98-1.03) | 1.00(0.93-1.08) | 0.99(0.93-1.06) | 1.01(0.97-1.04) |  |
| Age group | 18 - 54 | Ref | Ref | Ref | Ref |  |
|  | 55-64 | 1.17(1.09-1.26 | 1.1(0.9-1.33) | 1.1(0.95-1.29) | 1.03(0.98-1.07) |  |
|  | 65-74 | 1.22(1.14-1.29) | 1.14(0.96-1.35) | 1.16(1.01-1.33) | 1.02(0.97-1.07) |  |
|  | 75-84 | 1.20(1.12-1.27) | 1.23(1.04-1.45). | 1.18(1.03-1.36). | 1.05(1.00-1.11) |  |
|  | 85+ | 1.19(1.12-1.27) | 1.23(1.03-1.46). | 1.21(1.05-1.39)* | 0.98(0.9-1.07) |  |
| Ethnicity | Ethnicity | Ref | Ref | Ref | Ref |  |
|  | Black/Mixed/Others | 0.93(0.82-1.05) | 0.71(0.51-0.99) | 1.04(0.88-1.22) | 1.13(0.97-1.31) |  |
|  | British(White) | 0.97(0.89-1.05) | 0.87(0.72-1.03) | 0.95(0.83-1.08) | 1.01(0.89-1.15) |  |
|  | Irish(White) | 1.07(0.95-1.21) | 0.84(0.6-1.19) | 1.16(0.99-1.36) | 1.03(0.86-1.25) |  |
|  | Not Stated/Not known | 0.9(0.82-0.99) | 0.75(0.59-0.95). | 0.87(0.73-1.03) | 0.97(0.84-1.12) |  |
|  | Ethnicity Others(White) | 0.99(0.89-1.1) | 0.79(0.53-1.17) | 1.07(0.89-1.27) | 0.95(0.79-1.14) |  |
| Number of Contributory causes of death | 0 | Ref | Ref | Ref | Ref |  |
|  | 1 | 1.02(0.98-1.06) | 1.04(0.92-1.17) | 1.02(0.91-1.15) | 1.01(0.96-1.07) |  |
|  | 2 | 1.02(0.98-1.06) | 1.04(0.92-1.19) | 1.08(0.96-1.21) | 1.00(0.95-1.06) |  |
|  | 3 | 1.04(0.99-1.08) | 1.00(0.86-1.15) | 1.06(0.94-1.2) | 1.01(0.95-1.07) |  |
|  | 4+ | 0.97(0.93-1.01) | 0.99(0.87-1.14) | 1.05(0.93-1.18) | 0.96(0.91-1.01) |  |
| Socioeconomic status | 1 (Most Deprived) | Ref | Ref | Ref | Ref |  |
|  | 2 | 0.96(0.93-1) | 1.09(0.97-1.22) | 0.98(0.9-1.07) | 0.99(0.95-1.03) |  |
|  | 3 | 0.98(0.95-1.02) | 1.04(0.91-1.19) | 1.00(0.91-1.1) | 0.99(0.95-1.04) |  |
|  | 4 | 0.96(0.93-1) | 1.08(0.95-1.22) | 0.94(0.84-1.05) | 1.01(0.95-1.06) |  |
|  | 5 | 0.97(0.93-1.01) | 1.08(0.94-1.23) | 1.01(0.91-1.12) | 0.96(0.9-1.03) |  |
| Settlement | Rural | Ref | Ref | Ref | Ref |  |
|  | Urban | 1.01(0.97-1.06) | 1.03(0.9-1.17) | 1.04(0.91-1.21) | 1.06(0.98-1.16) |  |
| Underlying causes of death | Cancer | Ref | Ref | Ref | Ref |  |
|  | CBDs | 0.88(0.83-0.93) | 0.73(0.58-0.92)* | 0.99(0.87-1.13) | 0.77(0.69-0.86) |  |
|  | COPDs | 1.02(0.97-1.06) | 1.10(0.97-1.25) | 1.05(0.94-1.18) | 0.91(0.86-0.96) |  |
|  | CVDs | 0.85(0.81-0.9) | 0.96(0.84-1.1) | 0.88(0.78-0.99) | 0.85(0.8-0.91) |  |
|  | Neurological conditions | 1.10(1.04-1.17) | 0.91(0.68-1.22) | 0.83(0.61-1.12) | 0.72(0.48-1.06) |  |
|  | Other Deaths | 0.93(0.9-0.96) | 0.91(0.83-1) | 0.91(0.84-0.99) | 0.9(0.86-0.93) |  |
| Region | East Midlands | Ref | Ref | Ref | Ref |  |
|  | London | 1.05(1.00-1.11) | 1.02(0.89-1.18) | 1.02(0.91-1.15) | 0.96(0.89-1.03) |  |
|  | Midlands | 1.00(0.96-1.05) | 0.99(0.87-1.13) | 0.95(0.84-1.07) | 1.00(0.94-1.06) |  |
|  | North-East & Yorkshire | 0.97(0.93-1.02) | 1.00(0.87-1.15) | 0.96(0.85-1.08) | 0.96(0.9-1.02) |  |
|  | North-West | 0.96(0.91-1.01) | 1.05(0.92-1.21) | 0.92(0.81-1.04) | 0.93(0.87-1) |  |
|  | South-East | 0.97(0.92-1.01) | 0.96(0.83-1.11) | 0.88(0.77-1.01) | 0.98(0.91-1.04) |  |
|  | South-West | 0.98(0.93-1.03) | 1.07(0.92-1.23) | 0.83(0.7-0.98) | 0.94(0.87-1.02) |  |
